# Green synthesis of zinc oxide nanoparticles using *Elaeagnus angustifolia* L. leaf extracts and their multiple in vitro biological applications

**DOI:** 10.1038/s41598-021-99839-z

**Published:** 2021-10-25

**Authors:** Javed Iqbal, Banzeer Ahsan Abbasi, Tabassum Yaseen, Syeda Anber Zahra, Amir Shahbaz, Sayed Afzal Shah, Siraj Uddin, Xin Ma, Blqees Raouf, Sobia Kanwal, Wajid Amin, Tariq Mahmood, Hamed A. El-Serehy, Parvaiz Ahmad

**Affiliations:** 1 Department of Plant Sciences, Quaid-I-Azam University, Islamabad, 45320 Pakistan; 2 Department of Botany, Bacha Khan University, Charsadda, Khyber Pakhtunkhwa Pakistan; 3 Department of Biological Sciences, National University of Medical Sciences, Rawalpindi, Pakistan; 4 School of Life Sciences, School of Computer Science and Technology, Tiangong University, Tianjin, 300387 China; 5 Riphah International University, Sector I-14 Campus Hajj Complex Islamabad, Rawalpindi, Pakistan; 6 Department of Zoology, Rawalpindi Women University, Rawalpindi, 46000 Pakistan; 7 Department of Immunology and Genomic Medicine, Graduate School of Medicine, Kyoto University, Kyoto, Japan; 8 Department of Zoology, College of Science, King Saud University, Riyadh, 11451 Saudi Arabia; 9 Botany and Microbiology Department, College of Science, King Saud University, Riyadh, Saudi Arabia; 10 Department of Botany, S. P. College, Srinagar, Jammu and Kashmir 190001 India

**Keywords:** Biochemistry, Plant sciences

## Abstract

Due to their versatile applications, ZnONPs have been formulated by several approaches, including green chemistry methods. In the current study, convenient and economically viable ZnONPs were produced using *Elaeagnus angustifolia* (EA) leaf extracts. The phytochemicals from *E. angustifolia* L. are believed to serve as a non-toxic source of reducing and stabilizing agents. The physical and chemical properties of ZnONPs were investigated employing varying analytical techniques (UV, XRD, FT-IR, EDX, SEM, TEM, DLS and Raman). Strong UV–Vis absorption at 399 nm was observed for green ZnONPs. TEM, SEM and XRD analyses determined the nanoscale size, morphology and crystalline structure of ZnONPs, respectively. The ZnONPs were substantiated by evaluation using HepG2 (IC_50_: 21.7 µg mL^−1^) and HUH7 (IC_50_: 29.8 µg mL^−1^) cancer cell lines and displayed potential anticancer activities. The MTT cytotoxicity assay was conducted using *Leishmania tropica* “KWH23” (promastigotes: IC_50_, 24.9 µg mL^−1^; and amastigotes: IC_50_, 32.83 µg mL^−1^). ZnONPs exhibited excellent antimicrobial potencies against five different bacterial and fungal species via the disc-diffusion method, and their MIC values were calculated. ZnONPs were found to be biocompatible using human erythrocytes and macrophages. Free radical scavenging tests revealed excellent antioxidant activities. Enzyme inhibition assays were performed and revealed excellent potential. These findings suggested that EA@ZnONPs have potential applications and could be used as a promising candidate for clinical development.

## Introduction

For the past two decades, extensive research efforts have been made towards the preparation of cost-effective and eco-friendly nanostructured materials in the research fields of science, engineering and biotechnology^1,2^. Due to the high ‘surface-to-volume ratio’, nanoparticles (NPs) show unique and fascinating features such as optical, catalytic, size, shape, self-assembly and conductivity features^3^. Nanoparticles (NPs) are typically clusters 1–100 nm in size. Among the widely studied NPs, ZnONPs have gained significant attention and possess notable benefits in the production of pottery, transparent materials, elastic polymers, ointments, lubricants, dyes, adhesives and ceramic compounds^4,5^. ZnONPs have shown different biological and clinical applications, including antimicrobial, anticancer, antileishmanial, antioxidant, and enzyme inhibiting effects, as well as biocompatibility^6,7^. NPs are conventionally prepared by various physical and chemical methods (microwave irradiation, ultrasonication, sol–gel, wet impregnation, laser-vaporization routes, etc.^7–9^. Conventional methods of synthesizing nanostructured materials are costly, utilize toxic reagents, require complicated procedures and expensive equipment, consume a large amount of energy and time, employ noxious reducing agents, and require organic solvents and non-biodegradable stabilization agents and are thus environmentally harmful. The chemical reducing agents used cause environmental issues^2,10^. Therefore, many efforts have been made to develop an alternative method to formulate green and sustainable NPs and avoid the disadvantages of currently used procedures. The biosynthetic process is the most broadly recognized method due to several advantages: it is cost effective and eco-friendly, avoids several synthesis steps, requires no noxious chemicals, generates minimum waste, avoids harsh atmospheric conditions and does not require high temperatures, pressures or energy^11,12^. The biosynthesis of NPs utilizes environmentally friendly and cost-efficient reducing and stabilizing materials from plants, bacteria, fungi, yeasts, micro- and macro-algae and other natural resources without employing any toxic chemicals, thus reducing health and environmental risks^13,14^. Among the different natural resources, plant extracts are considered the most suitable candidates for the development of metal and metal oxide NPs due to the availability of numerous biomolecules, simple extract preparation, low cost, efficiency and rapid reaction rates. Additionally, plant extracts facilitate the control and precise synthesis of NPs with proper size and shape^15^. Hence, utilization of plant extracts for the synthesis of NPs is the best platform, being free from toxic chemicals. Natural plant extracts contain numerous beneficial phytomolecules that function as strong reducing, stabilizing and capping agents in the fabrication of NPs^16^. Other biological resources like bacteria and fungi may also be used, however they raise biosafety concerns. In addition, synthesis using microorganisms is challenging as it involve extensive and multiple steps for maintaining cell culture. Therefore the medicinal plants are preferred over microbial means for synthesis of nanoparticles^17–19^.

Numerous studies have concentrated on the green synthesis of ZnONPs using different medicinal plants^19–21^. The medicinal plant *E. angustifolia* used in the current study is native to Southern Europe and Western Asia and is also present in the Kashmir and Parachinar regions of Pakistan. The plant is locally known as Sanzala, belongs to the family Elaengnaceae, and is a nitrogen-fixing thorny shrub containing many phytochemical constituents: flavonoids, minerals, sugars, sterols, alkaloids, p-hydroxybenzoic, caffeic, protocatechuic acid, isorhamnetin, quercetin, kaempferol derivatives catechins, and vitamins (tocopherol, vitamin B1, vitamin C, α-carotene). The plant is used to alleviate pain and is used for the treatment of jaundice, rheumatoid arthritis, osteoarthritis, gastrointestinal problems, diarrhoea and asthma. According to traditional and ethnobotanical uses, various *E. angustifolia* extracts have shown significant antimicrobial, antioxidant, antiulcer, anti-inflammatory, and wound-healing potencies^22–25^. Nevertheless, there is no study available on the green synthesis of ZnONPs employing *E. angustifolia* natural leaf extracts. Therefore, the present study was undertaken to formulate ZnONPs employing *E. angustifolia* leaf extracts. Furthermore, the biogenic ZnONPs were characterized, and their structural, morphological and optical features were determined using various analytical tools, such as UV, XRD, FT-IR, EDX, SEM, DLS, TEM, and Raman spectroscopy. Finally, EA@ZnONPs were investigated for their multiple in vitro biological activities.

## Results and discussion

**Figure 1:**
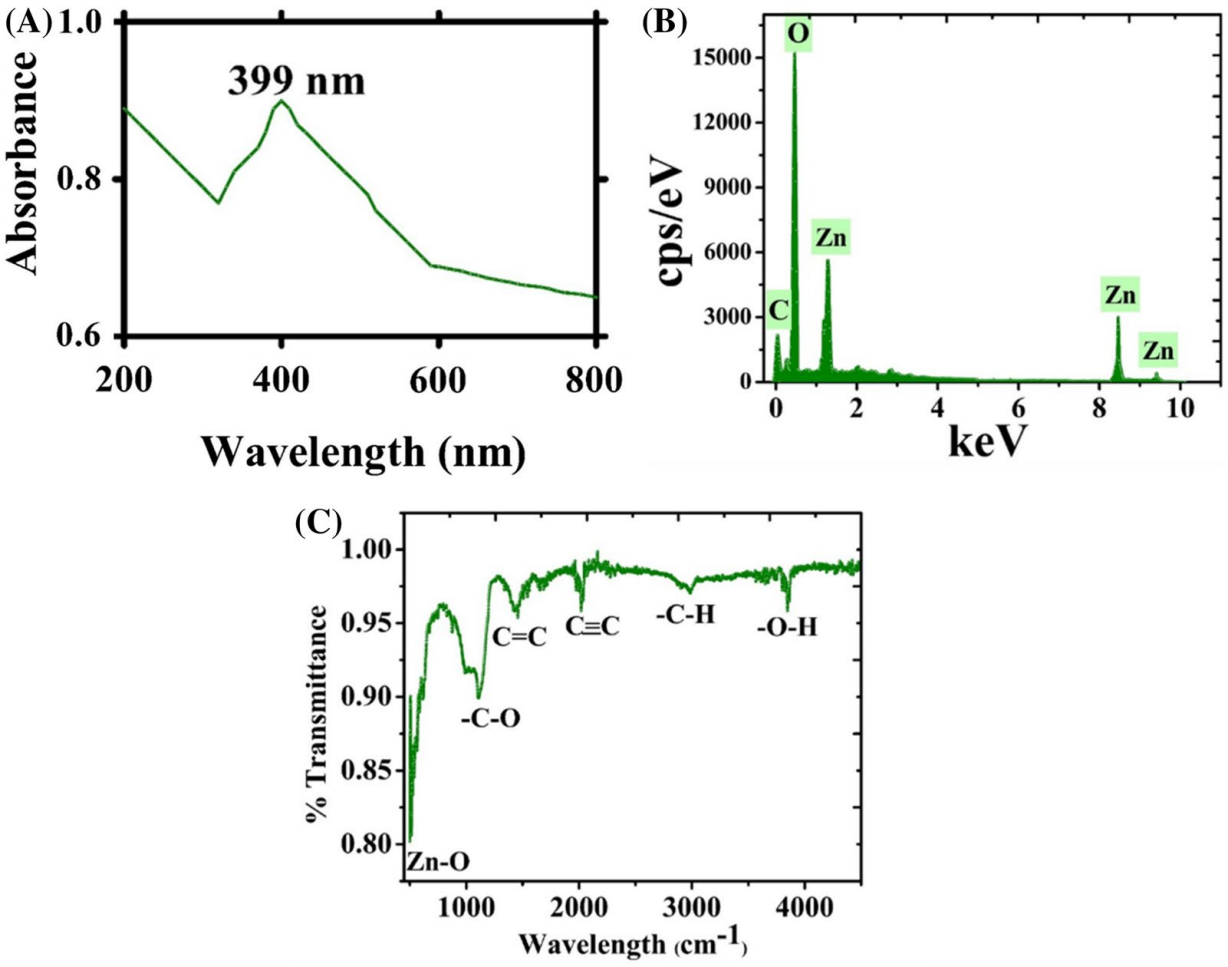
(**a**) UV visible spectroscopy, EDX and FTIR analysis of ZnONPs (**a**) UV; (**b**) EDX; (**c**) FTIR.

Although NPs can be prepared using multiple routes, green synthesis of NPs is superior to physical and chemical methods due to being simple, eco-friendly, cost efficient and free pf any toxic organic solvents or hazardous materials. The formation of NPs using physicochemical routes uses expensive and toxic chemicals, is environmentally toxic and requires high temperatures and pressures. These toxic chemicals sometimes remain adsorbed on the NP surface; as a result, these NPs cannot be utilized in biomedical settings. In the present study, we synthesized ZnONPs using *E. angustifolia*. This plant was selected due to containing multiple chemical components (flavonoids, minerals, sugars, sterols, alkaloids, p-hydroxybenzoic acid, caffeic acid, protocatechuic acid, isorhamnetin, quercetin, kaempferol derivatives, catechins, and vitamins (tocopherol, vitamin B1, vitamin C, α-carotene)^22–24^. These biomolecules play a potential role in the reduction, stabilization and capping of ZnONPs. Previously, ZnONPs have been prepared employing numerous medicinal plants^18^. In the present experiment, ZnONPs were synthesized using the leaf extract of *E. angustifolia*. During green synthesis of ZnONPs, the colour of the solution mixture of zinc nitrate hexahydrate and *E. angustifolia* leaf extract changed from light brown to yellowish black in colour. This colour change indicated the reduction of metallic zinc (Zn+) ions to zinc (ZnO) NPs. The obtained greyish material (assumed as ZnONPs) was further used for physical characterizations. The reduction of zinc ions was monitored in the reaction solution by measuring the UV-absorption of the solution via an optical UV-4000 UV–Vis spectrophotometer (Germany) at wavelengths of 200–800 nm. Figure 1a shows the absorption band at 399 nm (λ_max_), which is related to the SPR and indicates the formation of ZnONPs. The elemental composition and atomic content were confirmed using EDX. Figure 1b depicts the EDX spectrum of the synthesized ZnONPs, and the strong signal confirmed the synthesis of pure metallic ZnONPs. The signals relating to carbon and oxygen, which may have originated from bioactive compounds, function as stabilizers on the surface of ZnONPs. Moreover, no other peaks relating to any other elements apart from ‘Zn’ and ‘O’ were observed, confirming the phase purity of EA-ZnONPs. FT-IR analyses were performed to evaluate the role of the bioactive compounds in the aqueous *E. angustifolia* leaf extract in the fabrication of ZnONPs. The IR spectroscopic technique is also used to identify biomolecules in the field of natural products. The presence of different IR bands indicates various functional groups of biomolecules adsorbed onto the surface of ZnONPs, which confirmed the synthesis of ZnONPs by functioning as reducing and stabilization agents (Fig. 1c). For example, the peaks at a range of 498.25 cm^−1^ indicate Zn–O stretching vibrations; a band at 1106.12 cm^−1^ is related to C–O stretching, the peak at 1457.85 cm^−1^ corresponds to ‘C=C’, peaks at 2017.57 and 2980.72 cm^−1^ are related to C≡C and –C–H ‘stretching vibrations, and the peak at 3840.15 cm^−1^ can be ascribed to alcohols and phenols of OH stretching. These peaks indicate the availability of various biomolecules (flavonoids, minerals, sugars, sterols, alkaloids, p-hydroxybenzoic, caffeic, protocatechuic acid, isorhamnetin, quercetin, kaempferol derivatives, catechins, vitamins (tocopherol, vitamin B1, vitamin C, α-carotene)), which have been identified in previous studies^22–24^. According to earlier research work, the IR bands appearing between 470 and 800 cm^−1^ indicate Zn–O stretching vibrations^26,27^.

**Figure 2:**
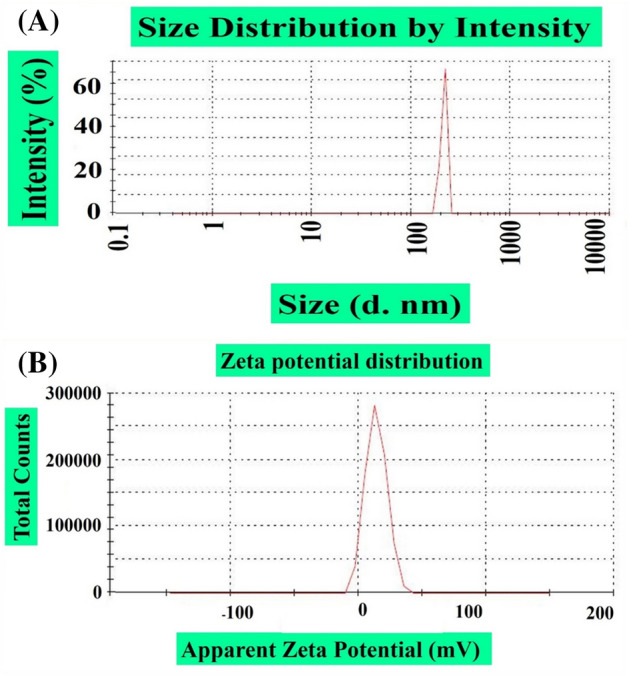
(**a**) Size distribution of greenly formulated ZnONPs (**b**) Zeta potential of greenly formulated ZnONPs.

The hydrodynamic size and stability of ZnONPs were acquired by DLS and zeta potential analysis. Furthermore, the zeta potential (ZP) is used to calculate the surface functionality and surface charge of NPs. The magnitude of the ZP of NPs determines particle stability, which ultimately determines their diverse applicability. NPs with a high ZP are particles with relatively high stability. In our experiment, the results demonstrated a particle size of 205.9 nm, a ZP of 13.8 mV and a PDI of 0.132 (Fig. 2a,b). Our DLS results of ZnONPs are in agreement with earlier reports using *R. virgata*-ZnONPs^5^. DLS analysis is mainly used to determine the size of particles in different suspensions. The mean hydro-dynamic particle diameter (d. nm) in water media determined the extent of particle aggregations^5,28^.

**Figure 3:**
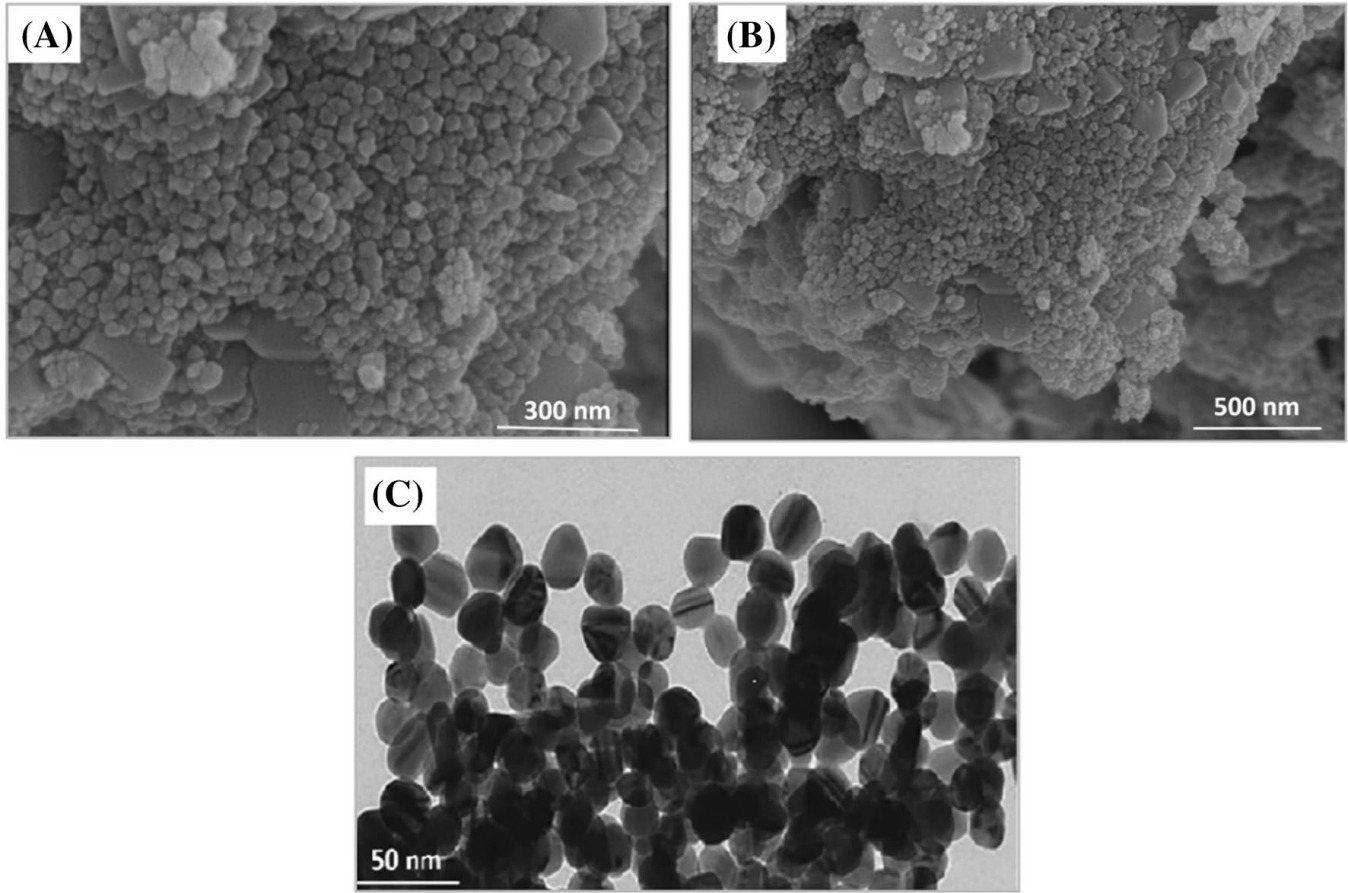
(**a**, **b**) SEM analysis of biogenic ZnONPs (**c**) TEM analysis of biogenic ZnONPs.

**Figure 4:**
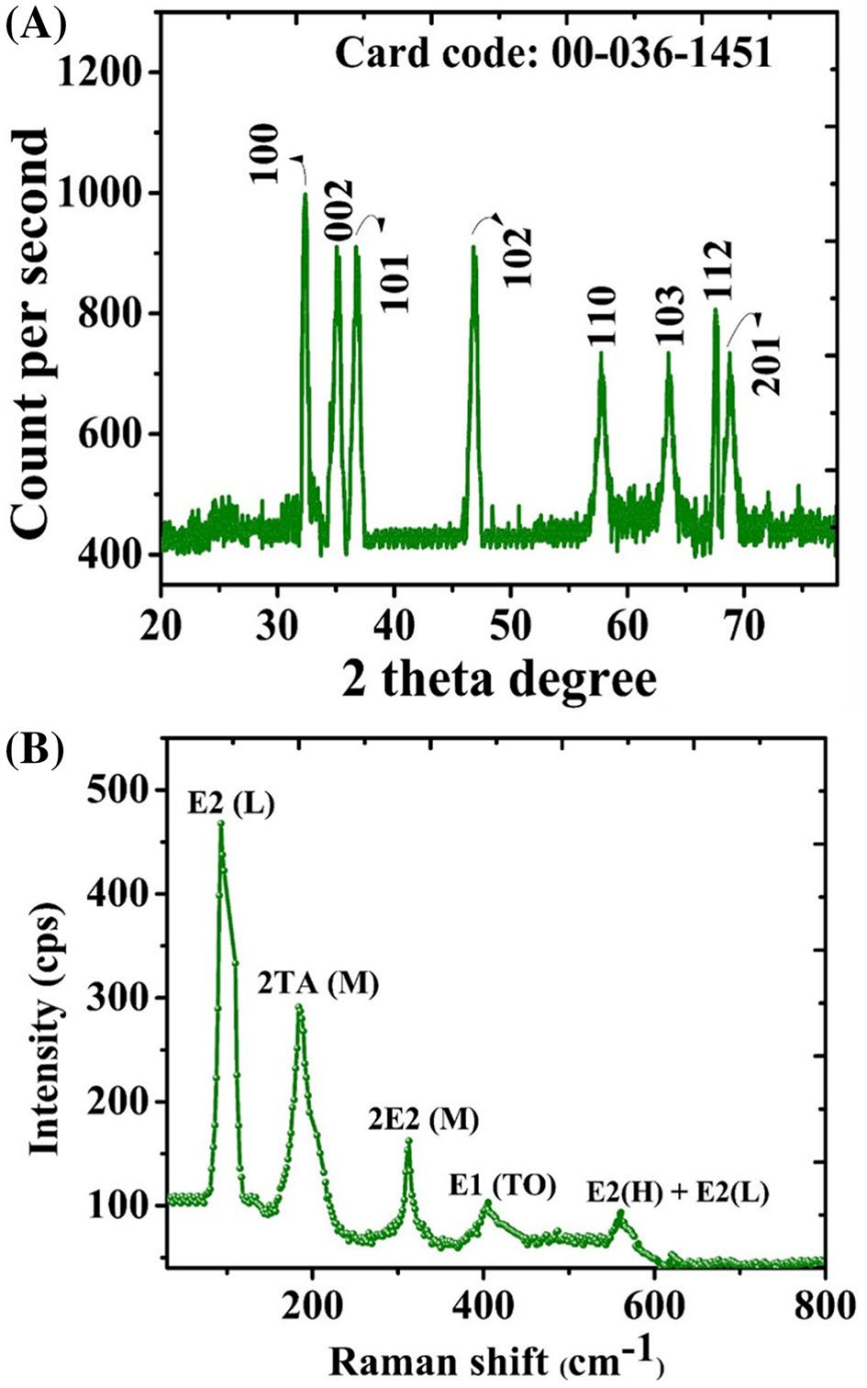
(**a**) X-ray diffraction and Raman analysis of ZnONPs (**a**) XRD; (**b**) Raman.

The morphology and shape of the green synthesized ZnONPs were investigated using scanning electron microscopy (SEM). The morphology of EA-ZnONPs is mostly spherical/agglomerated in shape, and typical SEM images are depicted in Fig. 3a,b. The surface morphology and size distributions of green synthesized ZnONPs were studied using TEM analysis. Spherical morphology was observed for ZnONPs. Our TEM size results of ZnONPs were in agreement with the calculated size of XRD using the Scherrer equation. The average size of ZnONPs is ~ 26 nm (Fig. 3c). Iqbal et al.^5^ and Abbasi et al.^6^ previously synthesized green ZnONPs from *G. wallichianum* and *R. virgata* leaf extracts and have shown similar results. Furthermore, XRD analyses were performed to determine the phase purity and crystalline nature of ZnONPs. The XRD patterns of ZnONPs are shown in Fig. 4a, which shows diffraction bands at 32.37 (100), 34.14 (002), 36.72 (101), 46.79 (102), 57.75 (110), 65.61 (103), 68.14 (112) and 69.94 (201) relating to fcc symmetry in the ZnONP crystalline lattice. In fact, broad peaks in the XRD pattern indicated the small size of the particle and determined the effect of experimental conditions, nucleation and growth of crystal nuclei. The synthesized EA@ZnONPs have a crystalline structure matching the gold standard by JCPDS (file no. -036-1451). The crystal size of ZnONPs was calculated using Scherrer's equation and was calculated as ~ 26 nm. Previously, green ZnONPs were synthesized using leaf extracts of *R. virgata* and *G. wallichianum*, and similar results were reported^5,6^. Raman spectroscopic analysis was performed to study the vibrational modes of *E. angustifolia*-ZnONPs. Figure 4b shows the positioning of major modes at 93.87 [E2 (L)], 185.09 [2TA (M)], 312.13 [2E2 (M)], 405.47 [E1 (TO)] and 558.97 cm^−1^ [E2(H) + E2(L)]. Intense peaks of ZnONPs indicated that the green NPs were rich in defects. The broad peak related to the antiferromagnetic nature and was related to the spinning of individual Zn++ ions. This antiparallel spin behaviour of ‘Zn++’ indicates that ZnONPs are nanoscale in nature (particle size: ~ 26 nm). The differences in Raman scattering peaks may be due to the relative position, size, intensity and effect of stress and strain^29^. Further Raman shifts indicated the purity of ZnONPs, and our spectroscopic results are in agreement with earlier reports using *G. wallichianum*@ZnONPs^6^.

### Evaluation of the biocompatibility potentials of ZnONPs

**Figure 5:**
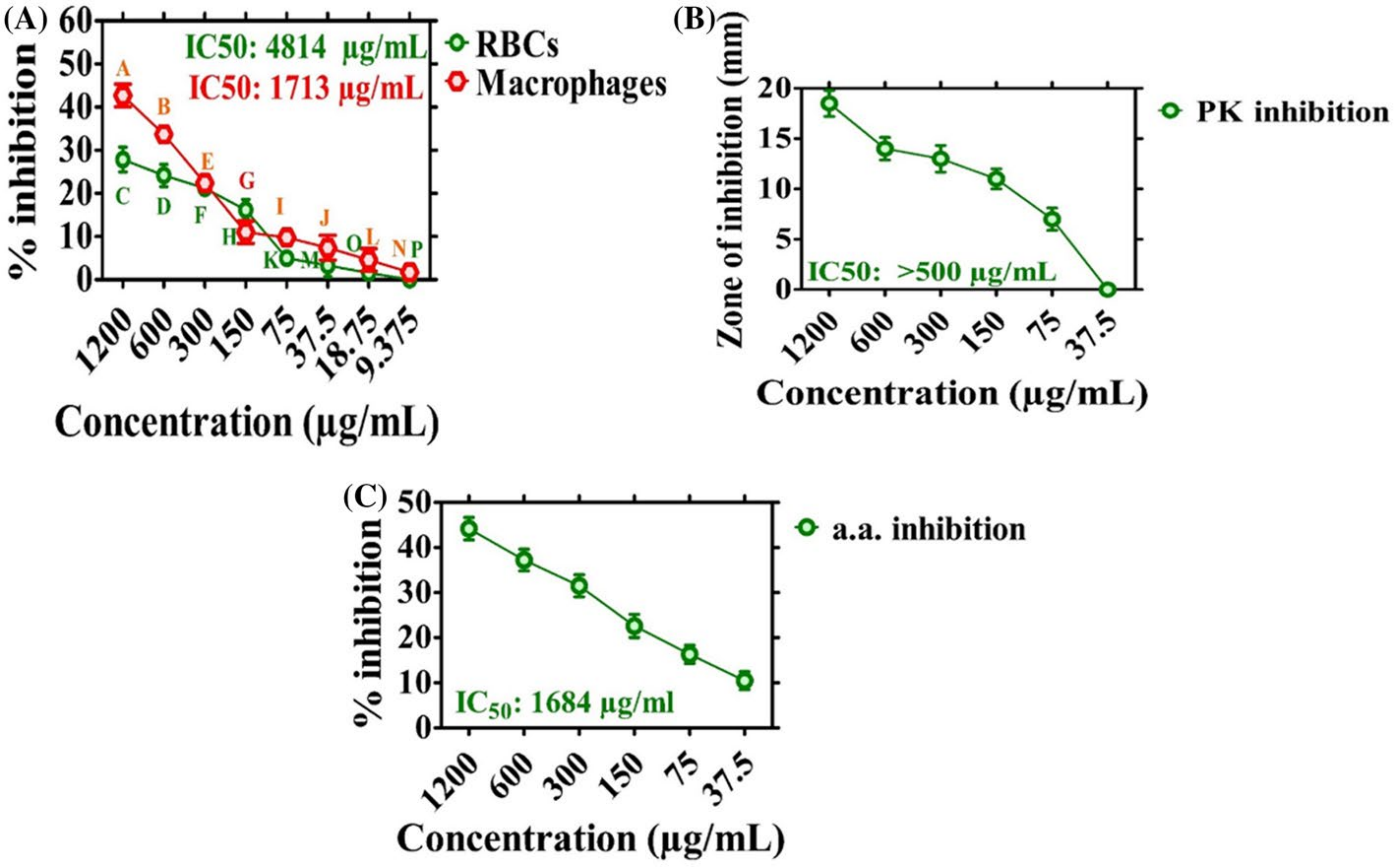
Biocompatibility assay, Protein kinase and Alpha amylase inhibition potential. Data represents the mean of three replicates and each letter indicates significance at P < 0.05. All means are significantly different from one another. (**a**) Biocompatibility assay using (RBCs and Macrophages); (**b**) protein kinase inhibition potential; (**c**) alpha amylase inhibition potential.

The biosafe and biocompatible nature of the synthesized ZnONPs were investigated. According to biosafety rules, biochemical substances with haemolysis > 5% are haemolytic, 2–5% are slightly haemolytic, and < 2% are not haemolytic^30,31^. If the tested sample (ZnONPs) shows haemolysis, it will rupture the erythrocytes and will result in haemoglobin release from erythrocytes. To evaluate the haemolytic nature of ZnONPs, erythrocytes were exposed to varying doses of ZnONPs (1200–9.375 µg mL^−1^), and concentration-dependent behaviour was observed. The % of haemoglobin release from RBCs was 27.83% at the highest ZnONP dose of 1200 µg mL^−1^ (Fig. 5a). Our experiment showed that EA-ZnONPs are non-toxic (biosafe) when used at low concentrations. Our EA-ZnONPs are similar to green ZnONPs synthesized using *G. wallichianum* and *R. virgata*^5,6^.

Additionally, the biocompatibility of ZnONPs was determined using HM cells. Confluent HM cells were seeded in 96-well plates containing RPMI medium and were grown for 24 h. In the next step, HM cells were exposed to various doses of EA-ZnONPs (1200–9.375 µg mL^−1^). MTT cell viability assays were established to investigate the biosafe nature of ZnONPs. Figure 5a indicates that green EA-ZnONPs at 1200 µg mL^−1^ inhibited HM cell growth by 42.7%, confirming their non-toxic behaviour. Our results of the test sample (ZnONPs) determined the concentration-dependent response of cell growth. Typically, HM cells have innate mechanisms and the potential to fight ROS generated from external sources. Different research reports have revealed that ROS are not toxic to erythrocytes and HM cells when they are present at relatively low concentrations unless their dose increases beyond the specified limit^32^. Our biocompatibility results of EA-ZnONPs are similar to the biocompatibility potential of ZnONPs synthesized using *R. virgata*^5^.

### Enzyme inhibition capacities of ZnONPs

*Elaeagnus angustifolia*-mediated ZnONPs were evaluated via the PK inhibition assay. Figure 5b shows the significant PK inhibition potentials of EA-ZnONPs using varying concentrations of the test sample ranging from 37.5–1200 μg mL^−1^. The results revealed moderate PK inhibition potential. Furthermore, ZIs were calculated to be 18.5 mm with an IC50 value ˃ 500 μg mL^−1^. The ZIs observed for the test sample were smaller than those obtained for the positive control (surfactin). The data revealed cell viability at a relatively low dose of ZnONPs. PK enzyme inhibition is considered a popular target for investigating the anticancer potential of chemical compounds. De-regulation of PK results in tumour progression^32,33^. Our data indicate that ZnONPs can inhibit the PK enzyme and therefore should be investigated for the treatment of cancer through PK inhibition. Our results are consistent with those of a previous study utilizing *G. wallichianum*-mediated ZnONPs^6^.

Furthermore, the enzyme inhibition potential of *E. angustifolia*-mediated ZnONPs was evaluated against the α-amylase enzyme. To reach this goal, alpha amylase enzyme was treated with different concentrations of ZnONPs (1200–37.5 µg mL^−1^). The EA-mediated ZnONPs were found to cause increased % inhibition (44.2%) at a concentration of 1200 µg mL^−1^ (Fig. 5c). However, percent inhibition potentially decreased with a decrease in ZnONP concentrations. Generally, moderate enzyme inhibition potentials were observed for the synthesized ZnONPs, and our present experimental results were in agreement with those of a previous study using *R. virgata*-ZnONPs^5^.

### Evaluation of antibacterial and antifungal assays

Worldwide, microbial resistance has grown at an alarming rate. To fight multi-drug-resistant (MDR) superbugs, biogenic ZnONPs were fabricated and used. ZnONPs have significant fungicidal and bactericidal activities against multi-drug resistant pathogens.

**Figure 6:**
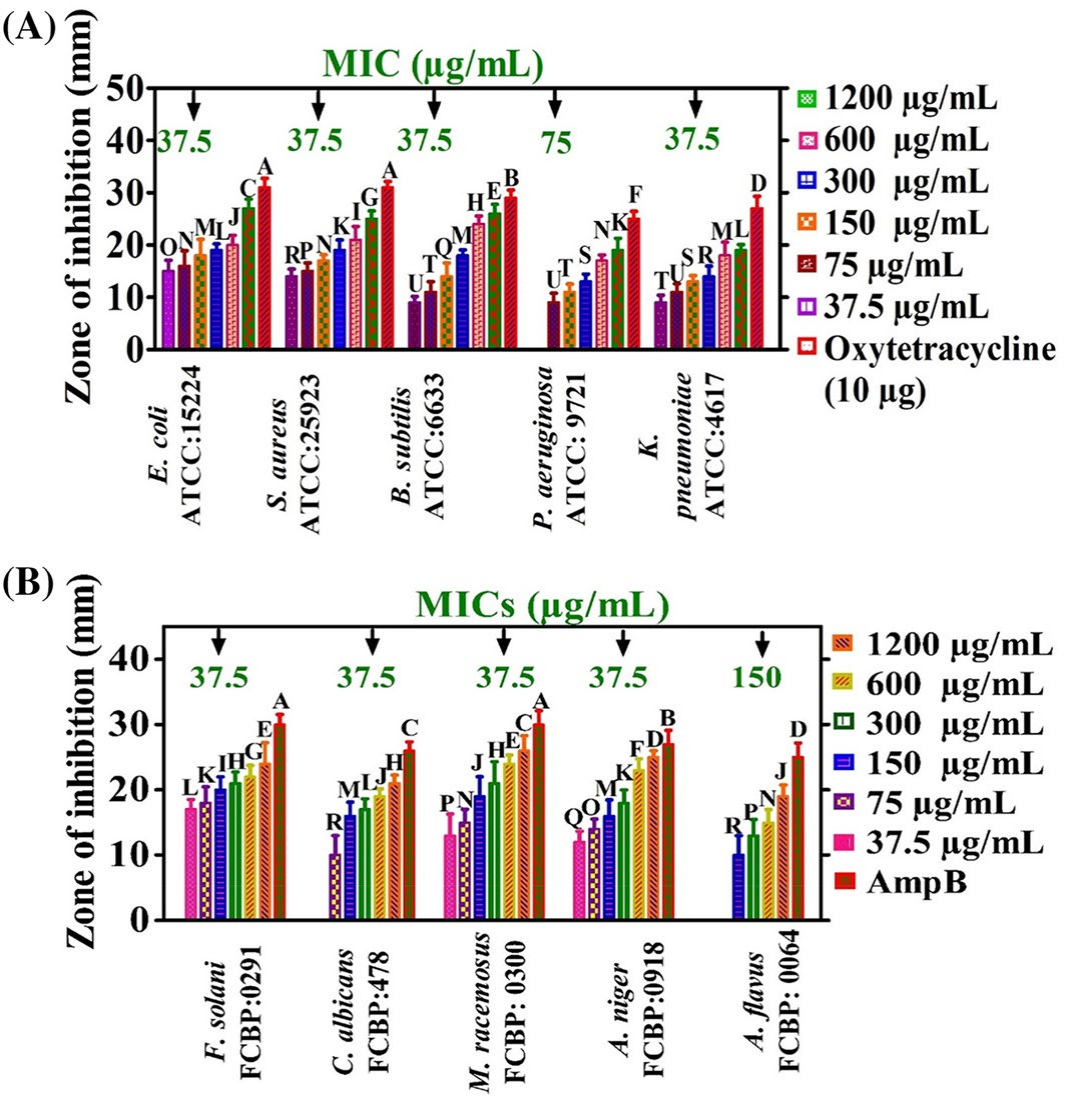
Antimicrobial activities of ZnONPs with MICs values. Data represents the mean of three replicates and each letter indicates significance at P < 0.05. There are 22 groups (A, B, etc.) in which the means are not significantly different from one another. Data represents the mean of three replicates (**a**) bactericidal activities; (**b**) fungicidal activities.

The antibacterial potentials of ZnONPs were investigated using various bacterial strains (BS) (*E. coli* ATCC 15224, *S. aureus* ATCC 25923, *P. aeruginosa* ATCC 9721, *K. pneumonia* ATCC 4617, and *B. subtilis* ATCC 6633) at concentrations ranging from 37.5 to 1200 µg mL^−1^. Most of the bacterial strains were observed to be susceptible to ZnONPs, and significant antibacterial activities against the tested BS strains were observed. Different MIC values were recorded for various BSs, such as 75 µg mL^−1^ (*P. aeruginosa*) and 37.5 µg mL^−1^ (*K. pneumoniae*, *E. coli*, *S. aureus*, and *B. subtilis*). Furthermore, *B. subtilis* was found to be the most susceptible strain with an MIC of 37.5 µg mL^−1^, while *P. aeruginosa* was the least susceptible strain (MIC: 75 µg mL^−1^). The antibacterial efficacy of ZnONPs against MDR human pathogens is shown in Fig. 6a. The bactericidal activity of ZnONPs showed that gram-negative BS are more susceptible to growth inhibition than gram-positive BS. Oxytetracycline was used as a positive control, and no single dose of ZnONPs showed stronger potential than the positive control. In summary, EA-ZnONPs showed dose-dependent antibacterial results, and our current experimental results are similar to those of previous studies of ZnONPs using medicinal plants^6,34^. The strong bactericidal potential of ZnONPs may be due to bioactive molecules adsorbed onto the NP surface. Further, research studies have demonstrated that bactericidal and fungicidal activities could be due to microbial cell-membrane perforations or they may be due to ROS produced by ZnONPs. Furthermore, these NPs can efficiently enter cell membranes through small pores present in the microbial cell membrane and may cause an imbalance of minerals and leakage of intracellular proteins and enzymes, ultimately resulted in cell growth inhibition and cell death^35^.

Extensive research has been performed on the antibacterial potencies of ZnONPs, while only limited research has been reported on the antifungal activities of ZnONPs. In this report, the antifungal properties of ZnONPs were explored against different fungal strains (FS) (*M. racemosus* FCBP 0300*, A. niger* FCBP 0918*, F. solani* FCBP 0291*, A. flavus* FCBP 0064*,* and *C. albicans* FCBP 478*).* To confirm the antifungal potentials of ZnONPs, fungal strains were treated with various doses of EA-ZnONPs (37.5–1200 µg mL^−1^). The antifungal potentials of ZnONPs are shown in Fig. 6b. Amp-B was used as a positive control to determine the inhibition potentials of ZnONPs. Our EA-ZnONPs revealed dose-dependent behaviour against various FSs. *A. flavus* was the least susceptible strain (MIC: 150 µg mL^−1^), while *A. niger* was the most susceptible strain (MIC: 37.5 µg mL^−1^). Previously, different dose-dependent antifungal assays were reported employing various fungal strains^5,36^, and the results are in match with our current ZnONPs.

### Anticancer potential of ZnONPs

**Figure 7:**
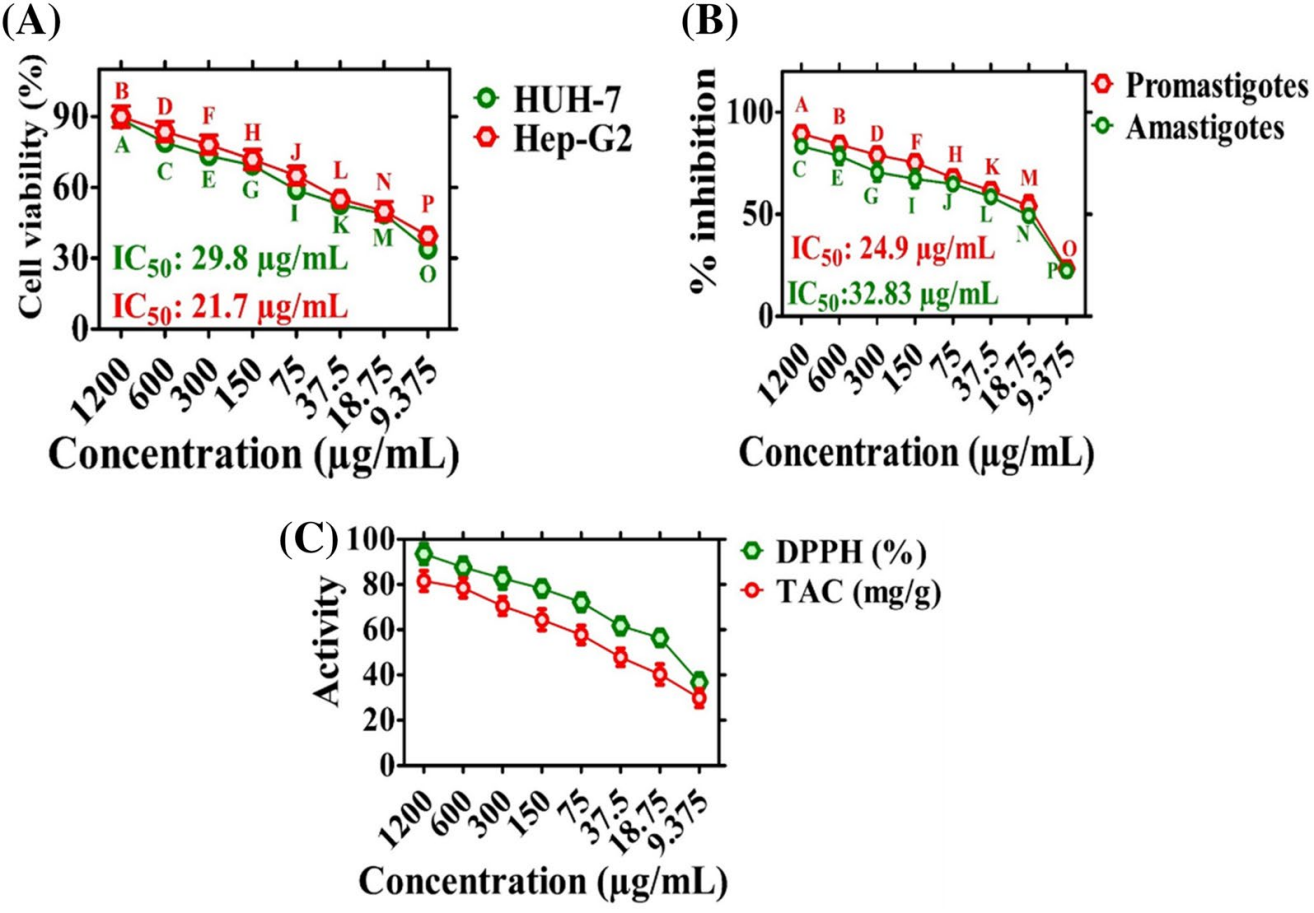
Anticancer, Antileishmanial and Antioxidant properties of ZnONPs. Data represents the mean of three replicates and each letter indicates significance at P < 0.05. All means in the data are significantly different from one another. (**a**) Anticancer potentials of ZnONPs against HUH-7 and HepG2; (**b**) antileishmanial potencies of ZnONPs; (**c**) antioxidant potential of ZnONPs Data represents the mean of three replicates.

In the current experiment, the anticancer properties of *E. angustifolia*-ZnONPs were explored against hepatocellular carcinoma cell lines (HUH-7 and HepG2: hepatocellular carcinoma using MTT assays. Cancer cell lines were exposed to varying doses of EA-ZnONPs (1200–9.375 μg mL^−1^), and a concentration-dependent response was observed. ZnONPs strongly reduced the metabolic activity of both cancer cell lines at varying concentrations. The metabolic activities of the cell lines decreased as the ZnONP concentration increased. The highest anticancer activity was recorded at 88.85% for HUH-7 cells and 89.98% for HepG2 cells at 1200 µg mL^−1^, and the anticancer activity decreased as the concentration of nanoparticles decreased. Furthermore, IC_50_ values were calculated for EA-ZnONPs, which were 29.8 µg mL^−1^ for HuH-7 and 21.7 µg mL^−1^ for HepG2. The anticancer activity even at low concentrations (9.375 µg mL^−1^) may be due to numerous biomolecules from leaf extracts adsorbed onto the surface of ZnONPs. The anticancer activity exhibited a dose-dependent response, and the results are shown in Fig. 7a. The reduction in metabolic activity indicated that EA-ZnONPs have strong anticancer activities. Our results of the present study are in agreement with those of previous reports employing *Euphorbia heterophylla* and *G. wallichianum*@ZnONPs^6,37,38^.

### Antileishmanial potential (ALP) of ZnONPs

The antileishmaniasis drug antimonial is a potent drug to treat leishmaniasis but has lost its biomedical potential, as *L. tropica* parasites have developed resistance against this drug. Thus, research scholars are making substantial efforts to develop an alternate strategy. Therefore, further comprehensive studies are required to develop novel and potential nanobiomaterials. Various nanomaterials have been reported for their ALP against *L. tropica* parasites^2,38^. However, biogenic EA-ZnONPs are rarely studied to establish their cytotoxic potential. The antileishmanial activity of ZnONPs was evaluated using an MTT cytotoxicity assay against *L. tropica* parasites. *L. tropica* parasites were treated with various concentrations of EA-ZnONPs ranging from 9.375 to 1200 µg mL^−1^. Figure 7b shows that the antileishmanial potential of EA-ZnONPs exhibited dose-dependent behaviour. The EA-ZnONPs showed potential results against *L. tropica* promastigotes (IC_50_: 24.9 μg mL^−1^) and amastigotes (IC_50_: 32.83 μg mL^−1^). In the current experiment, our results for ZnONPs were similar to those of a previous report utilizing *G. wallichianum*-ZnONPs^5^.

### Analysis of antioxidant activities

Figure 7c shows the antioxidant activities of *E. angustifolia*-mediated ZnONPs. DPPH (2,2-diphenyl-1-picrylhydrazyl) free radical scavenging activity (FRSA) was used to assess the occurrence of radical scavengers (antioxidant species) adsorbed on ZnONP surfaces. The DPPH FRSA assay determined a percent FRSA potential of 93.51%, and our DPPH results were similar to those of previous reports using *G. wallichianum*-based ZnONPs^6^. ZnONPs also revealed total antioxidant capacity in terms of AA E mg^−1^, and the maximum TAC value was reported as 81.34 mg g^−1^ at 1200 µg mL^−1^. The TAC purpose was to assess the scavenging potency of reductones/antioxidants present on ZnONPs towards ROS species. Our results were consistent with those of previously synthesized ZnONPs^5^. In general, antioxidant potency revealed the availability of radical scavengers in EA-ZnONPs, which further promoted their stabilization^3,39,40^.

## Conclusions and future perspectives

In conclusion, this study demonstrated a facile and eco-friendly procedure for the formulation of ZnONPs employing an *E. angustifolia* leaf extract, which is free of toxicants and rich in phytochemicals. The actions of different chemical components in the leaf extract may lead to the fabrication of ZnONPs. The synthesized ZnONPs were thoroughly studied for their physical and chemical properties. The UV–Vis spectrum determined an absorbance band at 399 nm that confirmed the reduction of Zn+ metal ions to Zn0 NPs. The size of the prepared ZnONPs was ~ 26 nm with a spherical shape. Importantly, this study also highlighted multiple biological activities of ZnONPs and revealed excellent antibacterial, antifungal, anticancer, antioxidant, biocompatibility and enzyme inhibition potential. The results showed dose-dependent behaviour of the ZnONP potency. Thus, it was evident from the present study that biogenic ZnONPs can be used as potential biosafe candidates in different biological applications. In the future, more in vitro, in vivo and mechanistic studies are needed in different animal models to evaluate their nanopharmacological relevance in multiple bioactivities.

## Materials and methods

All methods were carried out following relevant guidelines and regulations, and the Bioethics Committee approved all experimental protocols for Scientific Research at Quaid-i-Azam University Islamabad.

### Plant material collection and preparation of leaf broth

The medicinal plant *Elaeagnus angustifolia* L. was collected from Parachinar, Pakistan following standard practice and permission was obtained. Our plant study complies with relevant institutional, national, and international guidelines and legislation. Further, the plant material taxonomically identified (authorization number: SAS-557) by a senior taxonomist Dr. Syed Afzal Shah, Professor Department of Biological Sciences, National University of Medical Sciences, Rawalpindi, Pakistan. Leaf material of *E. angustifolia* was separated, properly washed with deionized water, shade dried and powdered. Leaf powder was preserved in a dry, airtight bottle and kept away from direct sunlight. In a separate flask, 100 mL of distilled water was heated to 100 °C, and then 20 g of dried *E. angustifolia* leaf powder was added. In the next step, the reaction mixture was incubated at 80 °C for 3 h on a hot plate. The aqueous extract was filtered several times to remove residual waste and debris. Furthermore, plant extract centrifugation was performed at 5000 rpm for 20 min to remove all unconsolidated materials, and the resulting extract was stored at 4 °C for future use.

### Green preparation of ZnONPs with Elaeagnus leaf extract

**Figure 8:**
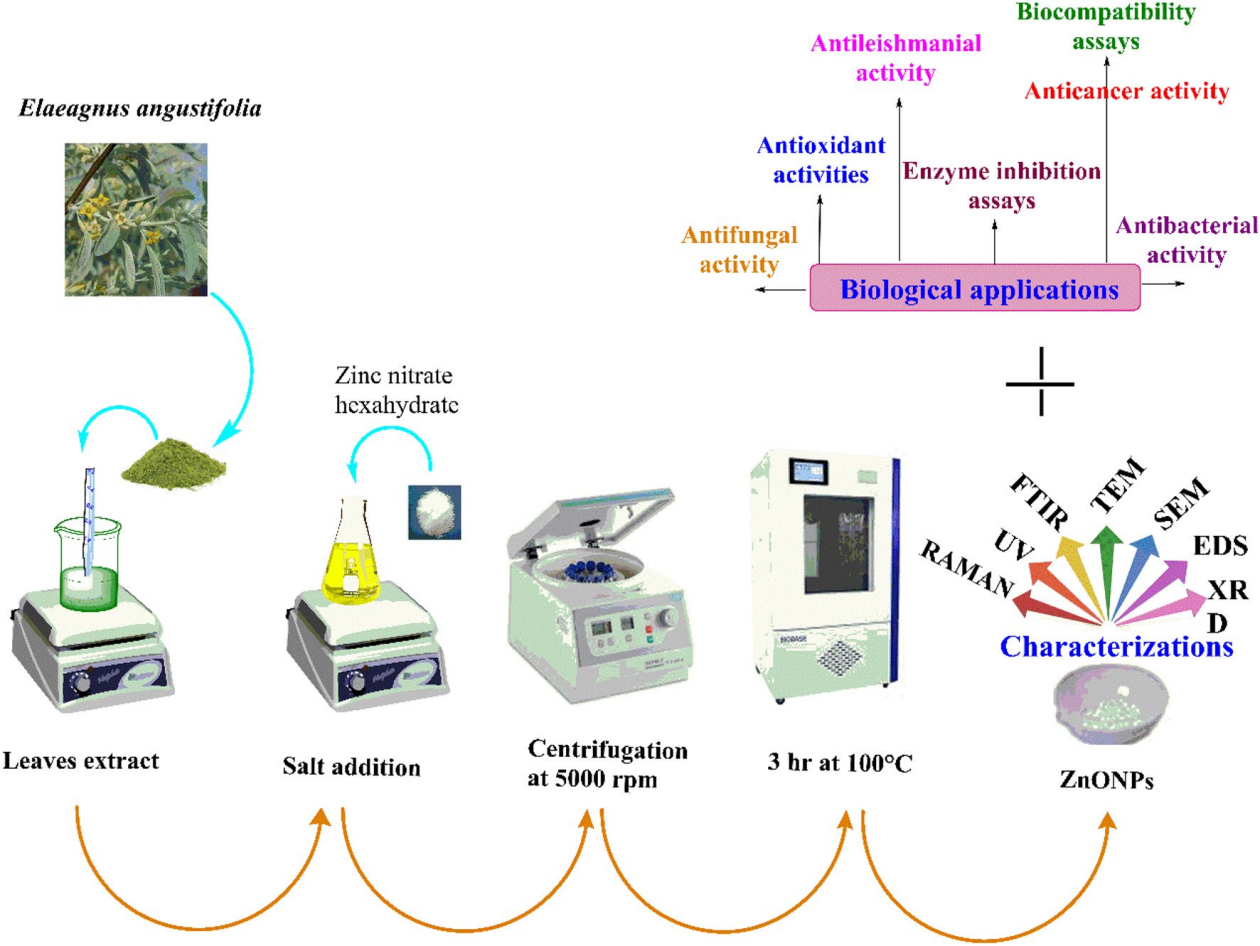
Study scheme depicting synthesis, characterization and biological applications of ZnONPs.

The biosynthesis of EA-ZnONPs was performed according to a previously established protocol with minor changes^6^. For the preparation of ZnONPs, 1 g of zinc-nitrate hexahydrate (Zn(NO_3_)_2_·6H_2_O) was allowed to react with 100 mL of *E. angustifolia* leaf extract. Furthermore, the mixture was continuously heated (60 °C/2 h). After 40 min, the solution colour turned yellowish black, indicating the synthesis of ZnONPs. The resultant solution was cooled at room temperature. The solution was centrifuged at 5000 rpm for 20 min, and the supernatant was removed and pellet at the bottom of falcon was retained. The obtained residue (assumed to be ZnONPs) was washed three times with dH2O to remove all uncoordinated biological material. In the next step, the product was washed with ethanol and dried in an oven at 100 °C for 3 h in Petri dishes. The FTIR spectra of newly synthesized nanoparticle was obtained to study the presence of functional groups adsorbed on the surface of NPs. Furthermore, the greenly synthesized nanoparticles were calcinated followed by different microscopic and spectroscopic techniques including UV, XRD, EDX, SEM, TEM, DLS and Raman. A schematic representation of EA-ZnONP synthesis is depicted in Fig. 8.

### Structural and morphological characterization

The EA-ZnONPs were extensively characterized via UV, XRD, EDX, FTIR, SEM, TEM, DLS, and Raman spectroscopy. In brief, the optical features and bioreduction of zinc ions to ZnONPs were studied via UV spectrophotometry (200–800 nm). The ZnONPs were centrifuged at 9000 rpm for 30 min and kept on a carbon film for XRD analysis (PANalytical XRD (Netherland). XRD analysis was performed to determine the crystal structure of ZnONPs. The vibrational properties and structural polarity of *E. angustifolia*-based ZnONPs were studied using Raman spectroscopy. FT-IR analyses were performed to detect the various functional groups involved in reducing and stabilizing ZnONPs. To remove non-binding *E. angustifolia*, synthesized ZnONPs were centrifuged at 8000 rpm/30 min, washed with dH2O and dried. The FT-IR spectra of ZnONPs were separately recorded in the region of 450–4500 cm^−1^. Further, the average hydro-dynamic particle diameter (d. nm), ζ‐potentials and PDI of EA-ZnONPs were determined utilizing a Malvern Zetasizer Nano. The particle size and shape of ZnONPs were investigated by TEM (transmission electron microscopy) and SEM by placing a drop of ZnONP suspension on a carbon-coated copper grid and drying it in air before microscopic analysis. EDX analysis was performed to study the elemental composition of the ZnONPs.

### Biological potentials of ZnONPs

Different biological activities have been performed to determine the biological potentials of synthesized nanoparticles using the relevant guidelines and regulations.

### Analyses of the biocompatibility potentials

The biosafe and biocompatible nature of ZnONPs were evaluated using human erythrocytes through a haemolytic assay^6^. The blood sample was willingly donated by one of the co-author Dr. Banzeer Ahsan Abbasi with informed consent. A total of 1 mL of fresh red blood cells was extracted and kept in an EDTA falcon tube. In the next step, erythrocytes were obtained by centrifuging at 12,000 rpm for 10 min. The upper phase was removed, and the pellet was washed several times with PBS. Furthermore, erythrocyte suspensions were prepared by loading erythrocytes (200 µL) in PBS (9.8 mL). The prepared erythrocyte suspension (100 µL) was treated with varying concentrations of test sample (ZnONPs). After treatment with ZnONPs, the reaction mixture was transferred into an incubator (36 °C/1 h), and centrifugation was performed at 12,000 rpm for 15 min. The upper phase was carefully collected and transferred to a 96-well plate, and haemoglobin release was calculated at 540 nm. Triton X-100 and DMSO were utilized as positive and negative controls to evaluate the biosafe nature. The % haemolysis produced by varying doses of ZnONPs was studied using the following equation:

$$ \% \;{\text{haemolysis}} = \frac{Sample\;abs - Negative\;control\;abs}{{Positive\;control\;abs - Negative\;control\;abs}} \times 100 $$

The biocompatible nature of ZnONPs was additionally confirmed using human macrophages (HMs) ^5^. To evaluate the non-toxic nature of ZnONPs, HM cells were sub-cultured in flasks containing RPMI medium supplemented with FBS (10%), Pen-Strep and Hepes. The flasks were transferred to a 5% CO_2_ incubator for 24 h for proper growth and attachment of HM cells. In the next step, ~ 4000 confluent cells well^−1^ were seeded into 96-well plates and exposed to various concentrations (1200–9.375 μg mL^−1^) of EA-ZnONPs. Finally, the % viability of HM cells exposed to varying concentrations of ZnONPs was recorded using the equation below:

$$ \% \;{\text{inhibition}} = \frac{{1{-}{\text{Absorbance}}\;{\text{of}}\;{\text{sample}}}}{{{\text{Absorbance}}\;{\text{of}}\;{\text{control}}}} \times 100 $$

### Enzyme inhibition potentials of ZnONPs

The protein kinase (PK) inhibition potency of the test sample was evaluated using a previously established protocol ^5^. To investigate the PK inhibition capacity of ZnONPs, SP4 minimal medium was prepared to achieve equal lawns of actinobacterium (Streptomyces 85E). One hundred microliters of Streptomyces 85E inoculum was taken from the standard culture through a pipette and was uniformly distributed on culturing plates using sterilized cotton swabs. Furthermore, sterilized 6 mm (millimetre) filter discs laden with varying concentrations (1200–37.5 µg mL^−1^) of ZnONPs were kept on the Streptomyces 85E-painted plates. Surfactin (positive) and DMSO (negative) were used as controls. In the next step, Streptomyces 85E plates were kept in an incubator at 30 °C for 72 h. After proper incubation time, varying inhibition zones (clear and bald zones) were observed, confirming the ZnONP inhibition property against Streptomyces 85E spore/mycelial formation. Finally, the zone of inhibition (ZI) was calculated in mm to determine PK inhibition potential.

The α-amylase inhibition potential of EA-ZnONPs was determined using a previously established protocol. Briefly, the reaction mixture was prepared by combining 45 µL of starch solution, 15 µL of test sample (EA-ZnONPs), α-amylase enzyme (30 µL) and FBS (20 µL). In the next step, 25 µL of HCl and iodine solution (95 µL) were added. Once the reaction mixture was prepared, it was further incubated at 50 °C for ~ 30 min. Acarbose and dH2O were used as positive and negative controls, respectively, to determine the α-amylase inhibition potential. The optical density (540 nm wavelength) was calculated using a microplate analyser, and the IC50 value was recorded. Finally, % inhibition was calculated employing the equation below:

$$ \% \;inhibition = \frac{{S\left( {ab} \right) - NC\left( {ab} \right)}}{{Blank \left( {ab} \right) - NC\left( {ab} \right)}} \times 100 $$

### Antibacterial activity of biosynthesized ZnONPs

The previously established DDM method was utilized to evaluate the bactericidal potential of EA-ZnONPs [10]. The antibacterial potential of ZnONPs was investigated using five different bacterial strains (*E. coli*, *S. aureus*, *P. aeruginosa*, *K. pneumoniae*, and *B. subtilis*). Before bactericidal activities were evaluated, the already available cultures were revived by sub-culturing the different bacterial strains into nutrient broth media, and flasks were incubated at 37 °C (rpm: 200, time: 24 h). Furthermore, 100 µL of standardized culture was loaded on agar plates, and uniform lawns were achieved with sterilized cotton swabs. The 6 mm filter discs were dispended with 30 µL of test sample (ZnONPs) from the already prepared dilution. In the next step, varying doses of ZnONPs (1200–37.5 μg mL^−1^) were studied using different bacterial strains. Oxytetracycline and DMSO were used as positive and negative controls, respectively. The plates were then kept in an incubator at 37 °C for 24 h and were periodically measured for ZI determination. Finally, MIC values were measured in millimetres.

### Antifungal activity of biosynthesized ZnONPs

The fungicidal potentials of ZnONPs were evaluated using various fungal strains (*M. racemosus*, A. niger, *F. solani*, *A. flavus*, and *C. albicans*). The disc-diffusion method (DDM) was established to investigate the fungicidal potencies. To study the fungicidal properties, nutrient broth medium was prepared and autoclaved. Furthermore, fungal strains were sub-cultured in flasks and transferred to a shaking incubator at 37 °C for 24 h. Furthermore, Sabouraud dextrose agar (SDA) medium was made and poured into Petri dishes. In the next step, Petri dishes were cotton swabbed (100 μL) with different pathogenic fungal strains to obtain equal lawns. Filter discs loaded with varying concentrations (1200–37.5 μg mL^−1^) of E. angustifolia-mediated ZnONPs were kept on Petri dishes. Amp B was loaded as a positive control, and DMSO was loaded as a negative control to determine the fungicidal potential of ZnONPs. The Petri dishes were transferred into an incubator at 37 °C for 24 h. After 24 h of incubation at 37 °C, the zone of inhibition (ZI) was measured in millimetres, and minimum inhibitory concentration (MIC) values were determined.

### Anticancer evaluation of ZnONPs

To further evaluate the cytotoxic potentials of the synthesized ZnONPs, an MTT cytotoxicity assay was performed using liver cancer cell lines (HepG2 and HuH7)^6,15,19^. These cell lines were acquired from American Type Culture Collection (ATCC), USA. For the determination of cytotoxicity, DMEM supplemented with FBS (10%) and Pen-Strep was used to culture liver cancer cell lines. Cancer cells (4000 cells well^−1^) were seeded into 96-well plates and incubated in a 5% CO_2_ incubator for 24 h at 37 °C to provide the proper culturing environment. After 24 h, plates were checked under a microscope for % confluency. In the next step, the cells were exposed to varying concentrations of the test sample (1200–9.375 μg mL^−1^) and incubated. The DMEM was removed, and fresh MTT solution (100 μL) was loaded, and the plates were kept in a 5% CO2 incubator at 37 °C for 3 h. DMEM was replaced with 100 μL of DMSO, and the platers were again placed into the incubator for ~ 30 min. The conversion of MTT solution to formazans by surviving cells was calculated at 570 nm. Untreated HepG2 and HuH7 cells were considered as controls, and anticancer potential was measured using the equation below:

$$ \% \;inhibition = \frac{1 - OD\;of\;sample}{{OD\;of\;control}} \times 100 $$

### Antileishmanial potentials of ZnONPs

The MTT cytotoxicity property of *E. angustifolia*-based ZnONPs was determined using the *Leishmania tropica* “KWH23 strain” (promastigotes and amastigotes)^2^. To evaluate antileishmanial potential, *L. tropica* parasites were cultured in a sterilized environment using MI-99 medium loaded with 10% FBS. In the next step, a 200 µL reaction mixture was prepared that contained 100 µL of standard culture, 50 µL of nanoparticle suspension, and fresh medium (50 µL). To evaluate the antileishmanial potentials of ZnONPs, Amphoterecin B and DMSO were used as positive and negative controls, respectively. Furthermore, *L. tropica* parasites in 96-well plates were exposed to varying concentrations of ZnONPs (1200–9.375 μg mL^−1^) and transferred into a 5% CO_2_ incubator (72 h at 24 °C). The absorbance was measured at 540 nm. After incubation, both living promastigote and amastigote parasites were counted, and IC_50_ values were recorded using the equation below:

$$ \% \;inhibition = \frac{1 - sample\;absorbance}{{absorbance\;of\;control}} \times 100 $$

### Assessment of the antioxidant properties of ZnONPs

Various spectrophotometric assays, such as DPPH free radical scavenging activity (FRSA) and TAC, were performed to confirm the radical scavenging potentials of EA-ZnONPs. Total antioxidant capacity (TAC) was determined by employing the phospho-molybdenum method^3,5,7,16,18,20^. TAC was determined to investigate the total antioxidant potential of the test sample (ZnONPs) at various concentrations ranging from 1200 to 9.375 μg mL^−1^. The incubation of EA-ZnONPs with molybdenum(VI) confirmed the occurrence of antioxidants adsorbed onto the ZnONP surface. Furthermore, different readings were recorded at 695 nm to evaluate its TAC potential. Ascorbic acid was used as a positive control, and DMSO was used as a negative control to better evaluate the TAC of the test sample.

For free radical scavenging activity (FRSA), 2.4 mg of DPPH was added to 25 mL of methanol to develop a free radical solution. To each well of the microplate, 20 μL of test sample was added, followed by the addition of 180 μL of the DPPH reagent solution, and the plate was incubated for 60 min (room temp) in the dark. In the next step, the prepared reagent was treated with varying doses (1200–9.375 µg mL^−1^) of ZnONPs and studied for FRSA. The absorbance of the reaction mixture was measured at 517 nm employing a microplate analyser to determine the presence of reductones. Finally, DPPH activity was calculated using the equation below:

$$ \% \;{\text{DPPH}}\;{\text{scavenging}} = 1 - \left( {\frac{Absorbance\;of\;sample}{{Absorbance\;of\;control}}} \right) \times 100 $$
